# Clinical and cost-effectiveness of vaginal pessary self-management compared to clinic-based care for pelvic organ prolapse: protocol for the TOPSY randomised controlled trial

**DOI:** 10.1186/s13063-020-04738-9

**Published:** 2020-10-08

**Authors:** Suzanne Hagen, Rohna Kearney, Kirsteen Goodman, Lynn Melone, Andrew Elders, Sarkis Manoukian, Wael Agur, Catherine Best, Suzanne Breeman, Melanie Dembinsky, Lucy Dwyer, Mark Forrest, Margaret Graham, Karen Guerrero, Christine Hemming, Aethele Khunda, Helen Mason, Doreen McClurg, John Norrie, Anastasia Karachalia-Sandri, Ranee Thakar, Carol Bugge

**Affiliations:** 1 Research Unit, Glasgow Caledonian University, Glasgow, UK; 2 The Warrell Unit, St. Mary’s Hospital, Manchester University Hospitals NHS Foundation Trust, Manchester Academic Health Science Centre, Manchester, UK; 3 University Institute of Human Development, Faculty of Medical Human Sciences, University of Manchester, Manchester, UK; 4 Yunus Centre for Social Business and Health, Glasgow Caledonian University, Glasgow, UK; 5 NHS Ayrshire & Arran, Crosshouse Hospital, Kilmarnock, UK; 6 School of Medicine, Dentistry & Nursing, University of Glasgow, Glasgow, UK; 7 NMAHP Research Unit, unit 13 Scion House, University of Stirling Innovation Park, Stirling, UK; 8 Health Services Research Unit (HSRU), University of Aberdeen, Aberdeen, UK; 9 Health Sciences & Sport, University of Stirling, Stirling, UK; 10 Patient and Public Involvement (PPI) representative, Dunlop, UK; 11 Department of Urogynaecology, NHS Greater Glasgow & Clyde, Glasgow, UK; 12 Aberdeen Maternity Hospital & Aberdeen Royal Infirmary, Grampian University Hospitals NHS Trust, Aberdeen, UK; 13 South Tees Hospitals NHS Foundation Trust, James Cook University Hospital, Middlesbrough, UK; 14 Usher Institute of Population Health Sciences and Informatics, College of Medicine and Veterinary Medicine, The University of Edinburgh, Edinburgh, UK; 15 Croydon Health Services NHS Trust, Croydon University Hospital, Croydon, UK

**Keywords:** Prolapse, Pessary, Self-management, Quality of life, Economic evaluation, Randomised controlled trial (RCT)

## Abstract

**Background:**

Pelvic organ prolapse (or prolapse) is a common condition in women where the pelvic organs (bladder, bowel or womb) descend into the vagina and cause distressing symptoms that adversely affect quality of life. Many women will use a vaginal pessary to treat their prolapse symptoms. Clinic-based care usually consists of having a pessary fitted in a primary or secondary care setting, and returning approximately every 6 months for healthcare professional review and pessary change. However, it is possible that women could remove, clean and re-insert their pessary themselves; this is called self-management. This trial aims to assess if self-management of a vaginal pessary is associated with better quality of life for women with prolapse when compared to clinic-based care.

**Methods:**

This is a multicentre randomised controlled trial in at least 17 UK centres. The intervention group will receive pessary self-management teaching, a self-management information leaflet, a follow-up phone call and access to a local telephone number for clinical support. The control group will receive the clinic-based pessary care which is standard at their centre. Demographic and medical history data will be collected from both groups at baseline. The primary outcome is condition-specific quality of life at 18 months’ post-randomisation. Several secondary outcomes will also be assessed using participant-completed questionnaires. Questionnaires will be administered at baseline, 6, 12 and 18 months’ post-randomisation. An economic evaluation will be carried out alongside the trial to evaluate cost-effectiveness. A process evaluation will run parallel to the trial, the protocol for which is reported in a companion paper.

**Discussion:**

The results of the trial will provide robust evidence of the effectiveness of pessary self-management compared to clinic-based care in terms of improving women’s quality of life, and of its cost-effectiveness.

**Trial registration:**

ISRCTN Registry ISRCTN62510577. Registered on June 10, 2017.

## Administrative information

Note: the numbers in curly brackets in this protocol refer to SPIRIT checklist item numbers. The order of the items has been modified to group similar items (see http://www.equator-network.org/reporting-guidelines/spirit-2013-statement-defining-standard-protocol-items-for-clinical-trials/).

**Table:** 

Title {1}	Clinical and cost-effectiveness of vaginal pessary self-management compared to clinic-based care for pelvic organ prolapse: protocol for the TOPSY randomised controlled trial
Trial registration {2a and 2b}.	ISRCTN Registry; ISRCTN62510577, registered on 06/10/2017
Protocol version {3}	Protocol Version 5. 25th July 2019
Funding {4}	This study is funded by the National Institute for Health Research Evaluation, Trials and Studies Coordinating Centre, Health Technology Assessment (NETSCC HTA) Programme (ref 16/82/01).
Author details {5a}	Suzanne Hagen^a^, Rohna Kearney^b^, Kirsteen Goodman^a^, Lynn Melone^a^, Andrew Elders^a^, Sarkis Manoukian^c^, Wael Agur^d^, Catherine Best^e^, Suzanne Breeman^f^, Melanie Dembinsky^l^, Lucy Dwyer^b^, Mark Forrest^f^, Margaret Graham, Karen Guerrero^g^, Christine Hemming^h^, Aethele Khunda^i^, Helen Mason^c^, Doreen McClurg^a^, John Norrie^j^, Anastasia Karachalia-Sandri^a^, Ranee Thakar^k^, and Carol Bugge^l^ ^a^ NMAHP Research Unit, Glasgow Caledonian University ^b^ The Warrell Unit, St. Mary’s Hospital, Manchester University Hospitals NHS Foundation Trust, Manchester Academic Health Science Centre; University Institute of Human Development, Faculty of Medical Human Sciences, University of Manchester ^c^ Yunus Centre for Social Business and Health, Glasgow Caledonian University ^d^ NHS Ayrshire & Arran, Crosshouse Hospital; School of Medicine, Dentistry & Nursing, University of Glasgow ^e^NMAHP Research Unit, unit 13 Scion House, University of Stirling Innovation Park, Stirling ^f^ Health Services Research Unit (HSRU), University of Aberdeen ^g^ Department of Urogynaecology, NHS Greater Glasgow & Clyde ^h^ Grampian University Hospitals NHS Trust, Aberdeen Maternity Hospital & Aberdeen Royal Infirmary ^i^ South Tees Hospitals NHS Foundation Trust, James Cook University Hospital j Usher Institute of Population Health Sciences and Informatics, College of Medicine and Veterinary Medicine, The University of Edinburgh ^k^ Croydon Health Services NHS Trust, Croydon University Hospital, Croydon ^l^ Health Sciences & Sport, University of Stirling
Name and contact information for the trial sponsor {5b}	University of Stirling Stirling FK9 4LA
Role of sponsor {5c}	The Sponsor played no part in study design; collection, management, analysis and interpretation of data; writing of the protocol and the decision to submit the report for publication.

## Background and rationale {6a}

Pelvic organ prolapse affects about 40% of women over 40 years of age [1], and the number of women affected is expected to rise [2]. Prolapse is categorised into different stages and types and affects women of varying ages. The distressing symptoms include a sensation of “something coming down” in the vagina, bladder, bowel and sexual problems and pelvic and back pain. These symptoms impact negatively on a woman’s quality of life [3].

Women presenting with prolapse are commonly offered conservative management (such as a vaginal pessary or pelvic floor muscle training) or surgery. There were over 28,000 hospital admissions in England in 2017/2018 related to female genital prolapse associated with approximately 42,000 bed days [4]. About 9.5% of women will undergo surgery for prolapse in their lifetime [5]. However, surgery is not always effective or durable with 30% of women requiring at least one further procedure [6]. With the high re-operation rates and the controversy surrounding surgery and the use of mesh implants, it is timely to consider the evidence supporting conservative options in more detail.

Currently, women who have prolapse of all types and stages can receive pessary treatment. Most commonly, women who use a pessary are over 60 years of age [7] and two thirds of women will opt to try a pessary when offered [8]. Although previous research indicates that the ring pessary is most commonly used in practice, a wide range of pessaries are fitted [9]. Hospital-based care remains the most common delivery mode for women who have a pessary with some community-based clinics and general practices also offering services. The most common service model for women is to return to a healthcare professional clinic to have their pessary removed and changed [7]. Most commonly, women attend a clinic appointment every 6 months for a pessary change, but time between changes does vary (3–12 months) [7]. It is not clear if pessaries would be used more often if pessary care was less reliant on follow-up appointments, allowing easier integration of pessary management with a woman’s lifestyle.

The largest UK-based observational study of pessary use reported that 86% of women successfully retaining a pessary at 4 weeks will continue to use a pessary at 5 years [10]. However, other studies have reported much lower continuation rates [11, 12]. Reasons for discontinuation of pessary use include developing complications such as bleeding or infection, dislike of the pessary changing procedure and inconvenience of attending appointments [13].

A UK multi-professional survey found that only 17% of clinicians offered women the option of self-managing their pessary [7]. This is a significant difference in practice compared with North America, where the majority of clinicians teach women pessary self-care [14]. The ongoing Cochrane review update has so far identified no completed trials including self-management for pessary in any comparison. Self-management focusses on actions that people undertake for themselves to manage their health and illness. In order to self-manage people need self-management support (actions taken to support people to self-manage, e.g. by healthcare professionals). Self-management has been shown to be effective in improving health outcomes such as quality of life in other conditions; e.g. condition-specific quality of life is improved for people with Chronic Obstructive Pulmonary Disease [15].

There is only one small (*n* = 88) non-randomised study that assesses self-management of vaginal pessaries [16]. Gains from self-management were reported in this study in that women reported higher levels of convenience, ability to access help, support and comfort than those having clinic management [16]. Women who were self-managing had one clinic appointment scheduled at 2 years, compared to health care professional clinic-based care where women attend a clinic every 4 to 6 months for pessary changes. Whilst these may be promising findings, there is an urgent need to robustly investigate whether pessary self-management is more clinically and cost-effective than standard pessary care. The TOPSY study aims to address this uncertainty to inform clinical practice.

## Objectives {7}

The aim of the TOPSY trial is to determine the clinical and cost-effectiveness of self-management of vaginal pessaries to treat pelvic organ prolapse, compared to clinic pessary care on condition-specific quality of life.

## Methods

### Trial design {8}

The TOPSY study includes a multicentre, parallel group, superiority randomised controlled trial (RCT), an internal pilot study and a nested process evaluation. The RCT and internal pilot will be described in further detail here, whilst the protocol for the process evaluation will be reported in a separate companion paper and will not be addressed further here.

The aim of the internal pilot is to ensure that the TOPSY trial can recruit, randomise and retain sufficient numbers of participants whilst delivering the intervention as planned. The internal pilot will aim to recruit 63 women across six centres (identified prior to the commencement of the study). The primary stop-go rules are detailed in the analysis section.

We will establish if pessary self-management is cost-effective compared to standard clinic-based pessary care by collecting cost and resource-use data for all participants using a combination of NHS data and participant-completed questionnaires. This is described in more detail in both the outcome section and the analysis sections.

### Study setting {9}

Healthcare providers with pessary care services (who have granted permission for the study to take place) will identify and recruit women (for more information on centres involved, please see https://w3.abdn.ac.uk/hsru/TOPSY/Public/Public/index.cshtml).

### Eligibility criteria {10}

Women will be eligible for inclusion if they are aged 18 or older, use a pessary of any type/material (except those that require more complex removal techniques such as Shelf or Gellhorn pessaries and those that must be self-managed such as cube pessary) and have retained the pessary for at least 2 weeks. Women will be ineligible if they: have limited manual dexterity that would affect their ability to remove and replace their own pessary; are judged by their healthcare team to have a cognitive deficit such that it is not possible to obtain informed consent or to self-manage; or are pregnant. The self-management teaching is only available in English; therefore, sufficient understanding of English language is required for participation.

### Recruitment {15}

Potential participants will be identified in the following ways:

1. Reviewing patient notes, clinic lists or caseloads to identify women who are currently using a pessary and could be approached;
2. At a pessary appointment when women attend for pessary review (existing users) or are fitted with a pessary for the first time (new users); and
3. Women who learn about the TOPSY study themselves (website, posters, word of mouth) and approach their centre or the trial office (this would be dependent on there being a TOPSY recruitment centre local to the women).

Women who are identified as potential participants through the mechanisms detailed above will be given a recruitment pack which contains an introductory letter, a participant information leaflet, an expression of interest form and a reply paid envelope. Women identified via patient notes, clinic lists or caseloads will have the recruitment pack posted to them by the “local TOPSY clinical team”. Women identified at their pessary appointment will be given the same recruitment pack in clinic. If time restraints in clinic mean that the local TOPSY clinical team are unable to fully discuss the study with the woman after giving out a pack, a follow-up call with the woman from a member of the research team at the centre delegated to do so can be arranged for an agreed time.

Once women have had enough time to make their decision, they can return the expression of interest form by post or in person to the local TOPSY clinical team to indicate if they are interested in participating or not. On receiving a positive expression of interest form, a member of the local TOPSY clinical team will discuss the study further with the woman and screen her for eligibility.

In addition, for women who are new pessary users (used a pessary for 3 months or less), eligibility screening will be finalised by a telephone call to assess if the pessary has been retained for at least 2 weeks. If the pessary has not been retained for 2 weeks, standard centre protocol would be followed for further pessary care. If women indicate that they remain interested in participating in TOPSY, eligibility will be reassessed once standard centre protocol is followed and the pessary has been retained for 2 weeks.

### Who will take informed consent? {26a}

If a woman is eligible and willing to take part, she will be asked to come to a baseline clinic appointment to provide written, informed consent for randomisation and completion of baseline questionnaires and demographic data (see “Outcomes” section for more information).

Informed consent procedures will ensure that women understand participation is voluntary and that participants can withdraw from all or any part of the research at any time without affecting their participation in other parts of the study, or their healthcare. Women may choose to withdraw from the treatment aspect of the study, but continue to provide data, for example by completing questionnaires. Where women cannot, or choose not to, continue to self-manage, this will be recorded and women, where willing to do so, will continue to complete questionnaires. If withdrawal occurs, the primary reason for withdrawal will be documented in the participant’s case report form (CRF), if possible. After full withdrawal, no further data will be collected from the participant but data collected up to that point will be analysed.

If a participant is randomised and then withdraws prior to any trial intervention being undertaken, for trial purposes the woman will continue to be included within her original allocated group, and if data are available, in the intention to treat (ITT) analysis. If women in the self-management group cross over to clinic-based care during the trial, they will follow the trial clinic-based care group protocol. A change of status form will be completed in all of the above examples to indicate the nature of the change of status and to monitor participant attrition rates. The Data Monitoring and Ethics Committee (DMEC) will review change of status information at an appropriately agreed frequency.

### Additional consent provisions for collection and use of participant data and biological specimens {26b}

The main trial consent asks participants if they would be willing to be contacted about the interviews (for the process evaluation part of TOPSY) or if they would be willing to have their teaching session or their 2-week follow-up call audio-recorded. They are also asked if they would be happy to be contacted in the future about research. Participants can say no to any of these questions and still take part in the TOPSY study.

### Assignment of interventions

The trial is supported by The Centre for Healthcare Randomised Trials (CHaRT; a fully registered UK CRN clinical trials unit in the Health Services Research Unit, University of Aberdeen). CHaRT will develop the data management system, a remote randomisation system, and will be responsible for ensuring the reliability of data at data-lock and compliance with the Research Governance Framework and Good Clinical Practice.

### Sequence generation {16a}

Randomisation will be minimised (naïve minimisation) by age (< 65/≥ 65 years), pessary user type (new user/ existing user) and centre.

### Concealment mechanism {16b}

This randomisation application will be available as an internet-based service, located within the TOPSY data management system.

### Implementation {16c}

A trained and delegated member of the local TOPSY research team will randomise women at each centre by accessing the data management system and entering the required information, which will generate the group allocation and display it on screen/and relay this information in an email.

### Who will be blinded? {17a}

The trial group to which women are allocated cannot be masked from the participants or the centre staff after randomisation has occurred. Blinding is therefore not possible*.*

### Procedure for unblinding if needed {17b}

Unblinding is not applicable.

## Intervention description {11a}

Self-management interventions are highly heterogeneous [14, 16–18], making identification of the effective component parts of an intervention difficult. However, based on evidence drawn from large scale self-management programmes, three tasks need to be achieved in order for individuals to self-manage [17]: medical management of the condition, role management and emotional management.

### Pessary self-management

To support a woman to achieve the three tasks needed for self-management, the intervention will be directed at three levels:

- At a service level to facilitate a supportive culture for a self-management treatment pathway.
- At a professional level to ensure that staff have the self-management teaching and support skills.
- At an individual woman level to ensure women can achieve the necessary tasks to self-manage.

### Supporting delivery of self-management at service and professional levels

At a service level, the TOPSY training team (a clinical co-applicant and the trial manager) will visit all trial centres and will discuss with staff the trial processes and the self-management protocol.

A training manual for those staff teaching women self-management has been developed: with Patient and Public Involvement (PPI) input (including a focus group with women from the Royal College of Obstetricians and Gynaecologists (RCOG) PPI group, Women’s Voices); through discussion with our clinical co-applicants (which includes urogynaecologists from across the UK, nurses and a physiotherapist); using International Consultation on Incontinence recommendations [19]; using best practice guidance from the self-management literature.

### Intervention components delivered to individual women

Women allocated to self-management will receive: a self-management teaching appointment, a self-management information leaflet, a 2-week follow-up telephone call, and a telephone helpline number/email address for their local clinical site.

Each woman in the self-management group will receive a 30 min, one-to-one self-management teaching appointment with an intervention healthcare professional (HCP) who has been trained in the pessary self-management intervention by the TOPSY training team. The intervention HCP is most likely to be a specialist nurse or physiotherapist, but may also be a urogynaecologist or general practitioner (GP). They have to be involved in pessary management as part of their clinical role to be eligible to teach women the intervention for the TOPSY study. The teaching appointment should take place within 4 weeks of the randomisation date. The self-management training manual specifies in detail the key components of the self-management intervention, facilitating standardisation of the self-management intervention across the centres. The key components as laid out in the training manual will be used by the intervention HCP when teaching women within the teaching appointment.

During the self-management teaching appointment, women will be given a self-management information leaflet containing written information on pessary self-management. The leaflet was initially developed as part of a previous non-randomised study [16] and is based on the viewpoints of, and feedback from, PPI representatives. The leaflet has undergone further development, drawing on the expertise of TOPSY PPI representatives, Women’s Voices (RCOG) focus group members and clinical co-applicants. The leaflet includes diagrams of various pessary types and pelvic floor anatomy, information about common complications and what to do if these are experienced. The same leaflet will be used across all centres.

### Strategies to improve adherence to interventions {11c}

During the self-management teaching session and reinforced in the self-management leaflet, women in the self-management group will be asked to remove, clean and re-insert their pessary at least once in the 2 weeks following the self-management teaching appointment. The woman will be telephoned 2 weeks after the appointment and asked if she has been successful in removing, cleaning and re-inserting her pessary. They will discuss any difficulties experienced. If the woman has not changed the pessary, the HCP will ask her to do so over the next week, and will call her again to check if this has been achieved. Where a woman has experienced difficulty that requires assessment by the HCP or where the woman has not changed the pessary by the time of the second phone call, she will be offered a second self-management teaching appointment. If, after this second appointment, the woman is unable to self-manage or does not wish to do so, she will be given the choice to transfer to clinic-based pessary care. All information on these interactions with women and any subsequent crossovers will be recorded in the study-specific CRF. Once it is clear that the woman has been able to remove and re-insert the pessary at least once, she will be asked to remove and re-insert the pessary at least once every 6 months. This information will be given as part of the self-management teaching appointment and is written into the information leaflet.

Women in the self-management group will receive a local telephone number and an email address to use to make contact with the intervention HCP at their centre if they experience any pessary problems or have questions (numbers of contacts received and details of reasons for calls will be recorded by the centre).

Women with PVC pessaries in both groups will receive a new PVC pessary every 6 months (women in the self-management group will either receive their new pessary by post or by picking up a prescription or some centres may give 2 extra pessaries at the baseline visit).

Women in the self-management group with silicone pessaries, which are more durable, will only have the pessary changed by request if required (e.g. if the pessary becomes damaged) and women with silicone pessaries in the clinic-based care group will have the pessaries changed as per local centre protocol. Self-management leaflets will include information about what women need to do if they require a new pessary.

Women in both trial groups will be asked to complete questionnaires every 6 months which will include questions regarding their patterns of pessary removal and re-insertion. At 18 months after randomisation, women in both groups will attend a clinic appointment which will include an examination of vaginal tissues, comparable to that carried out routinely in clinic-based pessary care (see below).

### Provisions for post-trial care {30}

Care will continue as normal for women in the standard care group. Local centres can provide training on self-management if this service is currently offered. For women who have been in the self-management group, it will be up to them and each local centre how often women are seen back in clinic. We will capture this information in the end of study case report form.

### Criteria for discontinuing or modifying allocated interventions {11b}

There are no special criteria for discontinuing or modifying allocated interventions. Participants may choose to revert back to standard care themselves for any reason or may choose to stop using a pessary.

### Relevant concomitant care permitted or prohibited during the trial {11d}

No special provisions.

### Explanation for the choice of comparators {6b}—clinic-based pessary care

Women will receive a clinic appointment for their pessary care according to the local management pathway. Content of appointments will follow local protocol which usually includes vaginal examination to remove the pessary, inspection of the vaginal tissues and insertion of a new pessary. Data on frequency of appointments and of pessary changes at appointments will be recorded in the CRF. Healthcare professionals who deliver standard pessary care at each centre will be interviewed as part of the process evaluation allowing variation in standard pessary care to be described.

## Recruitment and retention of study centres

Each collaborating centre will appoint a local intervention HCP as part of the local TOPSY research team who will be trained on the self-management intervention and who will keep regular contact with the local PI, with notification of any problems or unexpected developments. Each centre will have a centre initiation visit to ensure all study processes are in place before recruitment commences. The TOPSY Study Office will set up regular centre “forums” for all centres to “phone in” and discuss any problems experienced and share learning. Updates will be provided via quarterly newsletters. Centres having specific problems with recruitment and/or retention will be offered additional support either remotely or by an additional centre visit.

## Outcomes {12}

### Plans for assessment and collection of outcomes {18a}

Throughout the TOPSY study, data will be gathered from the women in the trial, and the TOPSY clinical research staff at each study centre. The outcome measures collected are described in the sections below and in Table 1.

**Table 1:**
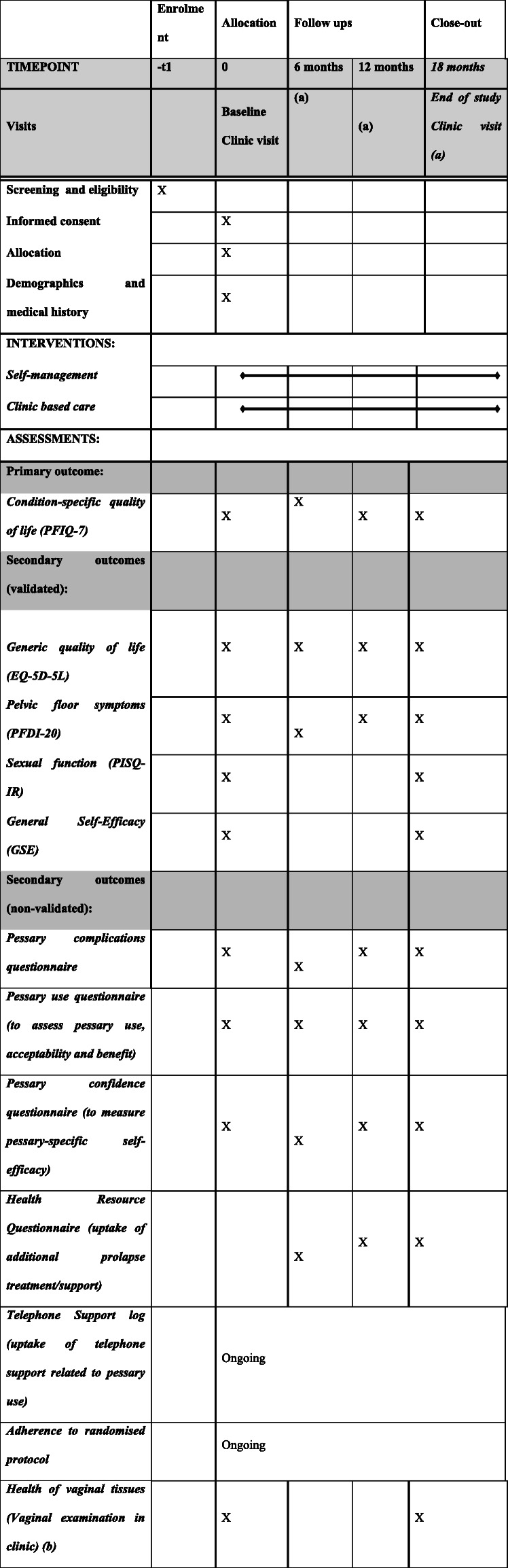
Item 13 in the SPIRIT checklist

(a) All women will complete their 6-, 12- and 18-month follow-up questionnaires via a paper questionnaire booklet or via a link to complete online (participant preference). Only women in the clinic-based care group will attend a clinic appointment as per the centre’s standard care

(b) Women in the clinic-based care group will have vaginal tissues assessed at each clinic appointment as per standard practice. Women in the self-management group will have their vaginal tissues assessed at baseline and 18-month appointments

### Primary outcome

The primary outcome, condition-specific quality of life at 18 months post-randomisation, will be measured via participant-completed questionnaires using the PFIQ-7. The PFIQ-7 [20] is a reliable, valid and responsive short form of the Pelvic Floor Impact Questionnaire (PFIQ) which measures condition-specific quality of life in women with pelvic floor disorders including urinary incontinence, prolapse and faecal incontinence. There are three subscales: urinary (UIQ-7), colorectal-anal (CRAIQ-7) and pelvic organ prolapse (POPIQ-7), with each sub-score ranging from 0 to 100 and a total score ranging from 0 to 300. Data will be collected at each time-point to allow repeated measures analysis of the PFIQ-7 scores.

### Secondary outcome measures

Several secondary outcomes will be collected as described below. Frequency of collection for each outcome is shown in Table 1.

Participants’ health-related quality of life will be measured by Euroqol (EQ-5D-5L) complementing the primary outcome measure of condition-specific quality of life, and also providing data for the analysis of cost-effectiveness (see section “Data collection for economic evaluation” for more information).

The severity of prolapse-related symptoms will be measured by PFDI-20. This was developed and validated in parallel with the PFIQ-7 [20]. It contains 20 questions about the presence of bladder, bowel and pelvic symptoms, and how bothersome these are.

Women’s sexual symptoms will be assessed by The Pelvic Organ Prolapse/Urinary Incontinence Sexual Questionnaire (PISQ-IR) [21].

A woman’s general self-efficacy (as a moderator of quality of life) will be measured by the General Self-Efficacy scale (GSE) [22].

#### Pessary complications

A new pessary questionnaire developed for TOPSY (with 15 possible complications of pessary use), developed based on the literature, PPI opinion and the team’s experiences in the pilot study, will be used to record women’s pessary complications (e.g. discharge, odour, pain, discomfort, bleeding). The questionnaire record will be used to collect the secondary outcome measure of pessary-related complications to report on the impact and safety of the trial interventions.

#### Pessary use

A new questionnaire (including eight questions) developed based on the literature, PPI consultation and the team’s experiences in the pilot study will be used to collect data on the pattern of a woman’s pessary use, including pessary continuation and perceived acceptability and benefit. This will include questions that ask women the following: whether or not they are still using a pessary as treatment for prolapse; when they last removed and re-inserted their pessary; reasons for pessary removal; interference of the pessary with everyday life; and if they find the pessary an acceptable treatment. Also included is a question adapted from the Patient Global Impression of Improvement (PGI-I) which will be used to assess perceived benefit of the pessary care regimens being evaluated. The PGI-I is a single-item tool asking the individual to rate the change in their condition since having treatment, which has been validated for urogenital prolapse [23, 24]. An amended version asking women to describe how they feel about their pessary care since taking part in the study will be used, with response options ranging from very much better to very much worse. Patterns of pessary use are used to measure impact, adherence and acceptability of the trial interventions.

#### Pessary confidence (to measure pessary-specific self-efficacy)

No suitable condition-specific measure exists; thus, questions relating to pessary self-efficacy were developed based on the guidance from Bandura [25]. These six questions have been developed with PPI representatives, PPI input, statistical input and clinical team members. We will use both the generic validated measure of self-efficacy (GSE) and the responses to the newly developed pessary-specific self-efficacy questions to measure self-efficacy and help us understand the influence it has as a moderating factor on quality of life.

#### Uptake of additional treatment for prolapse

As an indicator of intervention effectiveness, the uptake of other treatment for prolapse since the start of the study, or treatment awaited, will be recorded in participant questionnaires (e.g. surgery, pelvic floor muscle training, oestrogen, lifestyle advice). Women’s access to professional pessary-related support since starting the study will also be recorded (e.g. telephone support, hospital appointment, GP appointment). These data will be collected at all trial time-points to improve data quality as they rely on women recalling events occurring over a period of some months. Additional treatment will be described as part of the main trial findings to assist in understanding adherence and level of support women need, as well as being used as part of the cost-effectiveness analysis.

#### Uptake of telephone support related to pessary use

Using a Telephone Support Log Form, we will ask the intervention HCP who receives women’s calls to record frequency and details of all calls received to the telephone support line. There will be a question in the pessary complication questionnaire that asks *all* women if they required telephone support as some women in the clinic-based care group may also telephone for support from their local team. This will help understanding of adherence, effectiveness and level of support relating to the trial interventions.

#### Adherence to randomised protocol

Adherence to the self-management or clinic-based care protocol will be monitored throughout the trial. Monitoring will be via multiple data sources: questions within the pessary use questionnaire, telephone support contacts and health records. It will include crossover to the other trial group (i.e. self-managing women opting to move to clinic-based care). Clinic-based care women will not have access to the trial self-management teaching and support intervention, but they may choose to remove and replace their pessary at home and this will be recorded in the pessary use questionnaire.

#### Health of vaginal tissues

At baseline and 18 months, women will have a vaginal examination undertaken at the clinic by a healthcare professional to assess the health of the vaginal tissues and identify problems associated with pessary use, for example, tissue granulation or ulceration. Any findings will be recorded in the CRFs.

#### Biological specimens {33}

No biological specimens are collected as part of TOPSY.

### Data collection for economic evaluation

An economic evaluation will be conducted alongside the main trial. For both groups, the EQ-5D-5L will be completed as part of the participant questionnaires at baseline, 6, 12 and 18 months (http://www.euroqol.org/) to allow estimation of QALYs for a cost-utility analysis. In both trial arms, resource use will be captured by a combination of health data and participant-completed questionnaires. Questionnaires will be completed at 6, 12 and 18 months. Resource use related to appointments will be captured from patient case record forms at each appointment.

Overall costs will be estimated by multiplying resource use by unit costs obtained from the appropriate sources including trial-specific costs, NHS reference costs, Unit costs of Health and Social Care and the British National Formulary (BNF).

### Plans to promote participant retention and complete follow-up {18b}

Active measures to minimise loss to follow-up of women include:

1. Recording at the outset women’s email addresses and mobile phone numbers, their preferred method of contact (for follow-up contact) and their preferred method of completion of questionnaires. Questionnaires can be completed online (via an email link) or in paper format and returned by post.
2. Participants who do not return their questionnaires within 3 weeks will be sent up to three reminders using a variety of methods (post/email/ text message dependent on participants preferred method). The third reminder will be by telephone where the researchers will aim to gather, at a minimum, the primary outcome data during the call.
3. Response rates to the self-reported questionnaires will be monitored to ensure they remain above 80% (the level assumed in the sample size calculation). If response rates are seen to drop, the team will discuss appropriate actions with the project management group. Relevant action may include phone calls at different times of day or asking women to only complete the primary outcome measure.

### Data management {19}

All participants are given an individual study ID which will be used on all case report forms for that participant. Data will be entered into the secure database by the data coordinator based at the TOPSY study office at Glasgow Caledonian University. Local Centre staff will only enter the information required for randomisation (consent and eligibility information).

### Sample size {14}

The aim is to recruit a sample size sufficient to detect a 20-point difference in the PFIQ-7 score, which we consider to represent an important clinical difference (the potential range of the PFIQ-7 is 0 to 300). A sample size of 330 women (165 per group) is required to provide 90% power to detect a difference of 20 points in the PFIQ-7 score at 18 months, assuming a standard deviation of 50 based on previous studies [26, 27], two-sided alpha of 0.05 and 20% loss to follow-up. In order to detect this standardised effect size of 0.4 SDs (20/50 points), 132 women will need to be recruited per group, or 165 per group to allow for dropout.

### Stopping guidelines {21b}

Data from the internal pilot will be examined and the following stop-go rules will apply [28, 29].

- If the overall recruitment rate across pilot centres is 75% or more of the total expected recruitment (i.e. at least 47 out of 63), the trial will continue.
- If the recruitment rate is 50–75% (31–46 women), the trial will continue with a clear plan to overcome barriers to recruitment that is based on review of screening logs at centres, the trial protocol and the qualitative recruitment data (process evaluation).
- If the recruitment rate is 25–50% (16–30 women), screening logs, the protocol and the qualitative recruitment data (process evaluation) will be reviewed and the trial will only continue after discussion with and approval by NIHR HTA and with a clear plan to recruit within more centres and address the recruitment shortfall.
- Should recruitment be < 25% (15 women or less), we will enter into discussions with the funder but it is not expected the trial will progress. The decision to stop the trial will be made by the TSC and the funder.

In addition, we have set the following secondary targets:

- 40% of eligible new and 20% of eligible existing pessary users invited agree to randomisation;
- 60% of the pilot self-management women (*n* = 19 of 31 women randomised to self-management) still self-managing at 2-week telephone follow-up (i.e. have removed and re-inserted their pessary at least once).

### Statistical methods for primary and secondary outcomes {20a}

A single main analysis will be performed at the end of the trial when 18-month follow-up has been completed. The independent DMEC will review confidential interim analyses of accumulating data at its discretion but at least annually. All analyses will be conducted according to a pre-specified statistical analysis plan.

All outcomes will be described with the appropriate descriptive statistics: mean and SD for continuous outcomes (or medians and interquartile range for skewed data), and counts and percentages for dichotomous and categorical outcomes.

The main effectiveness analysis will be based on the ITT principle. The analysis of the primary outcome will estimate the mean difference (with 95% confidence intervals) in the PFIQ-7 score at 18 months between the self-management and standard care groups using a mixed effects repeated measures model (which assumes incomplete outcome data to be missing at random). The model will incorporate age (< 65/≥ 65) and pessary user type (new/existing) and baseline PFIQ-7 as fixed effects and participant and recruitment centre as random effects. Statistical significance will be at the 5% level.

Secondary outcomes will be analysed using an appropriate generalised linear model, for example binary logistic regression for dichotomous outcomes such as discontinuation with pessary (Y/N), and ordinal logistic regression for ordered categorical outcomes such as women’s global impression of improvement (PGI-I). All models will be adjusted for minimisation covariates (age, pessary user type and centre) and baseline score (where applicable).

#### Methods in analysis to handle protocol non-adherence and any statistical methods to handle missing data {20c}

The missing at random assumption for primary outcome data will be assessed further in sensitivity analyses. Treatment effects will be estimated under varying assumptions of data being missing not at random using pattern-mixture models. A complete case analysis will also be conducted.

Given the potential for crossover, we will conduct a secondary analysis of compliers to estimate the effect of receiving the self-management intervention, using complier average causal effect (CACE) estimators. The CACE analysis will take a maximum likelihood approach, which can assume incomplete data to be missing at random, and can be adjusted for covariates. This analysis will provide unbiased effect estimates of receiving the self-management intervention, which will complement the ITT effect estimates.

#### Methods for additional analyses {20b}

Subgroup analyses will be carried out within the following groups: age (< 65/≥ 65 years), hysterectomy (Y/N) and type of pessary user (new versus existing). Stricter levels of statistical significance (2P < 0.01) will be sought, reflecting the exploratory nature of these analyses. Heterogeneity of treatment effects amongst subgroups will be tested for using the appropriate subgroup by treatment group interactions [30].

### Economic analysis

#### Cost-effectiveness analysis

The primary analysis will be undertaken at 18 months from an NHS perspective. All costs and outcomes beyond 1 year will be discounted at 3.5% [31]. A broader perspective including women’s personal expenditures will be included in a sensitivity analysis. Incremental cost-effectiveness ratios (ICERs) will be computed by comparing the costs and outcomes of the self-management and clinic-based care trial groups. The difference in effectiveness will be expressed in terms of the change in score on the primary outcome measure PFIQ-7 (cost-effectiveness analysis). The difference in utility between the two groups will be expressed in terms of QALYs calculated using the UK value set for patient-reported EQ-5D-5L data [32]. This will be used in a cost-utility analysis to calculate the incremental cost per QALY gained*.*

#### Longer-term decision modelling

To examine the costs and outcomes of self-management compared to clinic-based care beyond the trial period, a decision analytic model will be developed. This will involve extrapolating data from the trial period and supplementing with additional data from the literature and other data sources as required. A 5-year time frame will be used and the care pathway over this period will be mapped out. We will incorporate data on the number of women who would want to self-manage, continued pessary use, continuation rates for self-management, complications and adverse events, conversion to surgery rates for both self-management and clinic-based care, health outcomes (prolapse and general quality of life outcomes), expenditure attending follow-up appointments in both groups, expenditure on replacement pessaries in both groups (type-dependent) and other (potentially rare) outcomes of interest that we would unlikely to see during the 18-month trial period (e.g. fistula). Using this model, we will perform cost-effectiveness analyses by synthesising trial data and data from other sources. Robustness of the results will be assessed through probabilistic sensitivity analyses. This will allow us to examine longer-term outcomes and cost-effectiveness under the presence of uncertainty.

### Interim analyses {21b}

A single main analysis will be performed at the end of the trial when the 18-month follow-up has been completed. The DMEC will review confidential interim analysis of accumulation data at its discretion but at least annually.

### Composition of the coordinating centre and trial steering committee {5d} and composition of the data monitoring committee, its role and reporting structure {21a}

An independent trial steering committee (TSC) will review the study on behalf of the sponsor and the funder. A separate and independent data monitoring and ethics committee (DMEC) will be convened. Both committees will have an independent chair. The DMEC will report to the TSC. During the period of recruitment to the trial, the DMEC will review a report on accumulating safety data at each meeting, together with other analyses that the committee may request.

The trial will also be overseen by a project management group (PMG; consisting of the grant applicants, the trial staff and 3 public and patient representatives).

### Frequency and plans for auditing trial conduct {23}

The TSC and DMEC will meet every 6 months during recruitment and then annually. The PMG will meet (via teleconference) every 6 to 8 weeks. The TOPSY study office will monitor the quality of the data returned by the study centres and action accordingly.

### Adverse event reporting and harms {22}

All women in the TOPSY study have had a vaginal pessary inserted. As a foreign body placed in the vagina, this is recognised as a potential cause of specific symptoms, e.g. bleeding and vaginal ulceration/erosion. Expected events arising from pessary treatment are noted below and thus will NOT be collected as adverse events but will be recorded:

- Granulation of vaginal tissue
- Involuntary expulsion of pessary
- Vaginal smell
- Vaginal discharge
- Bleeding during pessary change.

The questionnaires completed at the 6-, 12- and 18-month follow-up include a pessary complication questionnaire where women will indicate any complications they have experienced.

In the clinic-based care group, the local clinical TOPSY research team will ask about the occurrence of AEs/SAEs at every pessary follow-up appointment. Open-ended and non-leading verbal questioning of the participant will be used to enquire about AE/SAE occurrence. Participants will also be asked if they have been admitted to hospital, had any accidents, used any new medicines or changed medication regimens. If there is any doubt as to whether a clinical observation is an AE, the event should be recorded. Women in the self-management group are asked during the teaching appointment and advised in the information leaflet to call the telephone helpline if they experience any of the symptoms that may be indicative of an SAE/AE. The pessary complication questionnaire completed by all women at all time-points will also capture any adverse events experienced.

We have adhered to the new structured study protocol template which includes all SPIRIT headings and item identifiers.

### Dissemination plans {31a}

In addition to the main funding report, journal publications and conference presentations, we will make the training manuals and materials available online and training days will be arranged. Where possible our relevant patient and clinical representatives will be part of the dissemination activities (training days, presentations).

### Plans for communicating important protocol amendments to relevant parties (e.g. trial participants, ethical committees) {25}

Funders, sponsors and National Health Service Research & Development Offices will be notified routinely and appropriate approvals gained and communicated as required by them and by the trial sponsor.

### Plans to give access to the full protocol, participant level data and statistical code {31c}

The full Trial Protocol is available on the funder’s website (https://www.journalslibrary.nihr.ac.uk/programmes/hta/168201/#/). A second paper detailing the process evaluation of TOPSY has been submitted to TRIALS as a companion paper. Anyone interested in other data or documentation should contact the corresponding author.

## Discussion

Due to the anticipated rise in the prevalence of prolapse with an ageing population, there will be an anticipated increasing demand on pessary management services which will have increased cost implications across all NHS trusts. Some pessary management services in different regions across the UK offer women the option to learn how to self-manage their own pessaries but there is limited evidence to support this practice. There is currently no “gold standard” on how self-management is taught to women, no evidence on the support structures that should be available to support self-managing women or on how often women should be seen in clinic if self-managing. The TOPSY study is therefore crucial to evaluate the effectiveness of self-management on a woman’s quality of life and the potential impact on current and future NHS workload. TOPSY is the first trial of self-management in pelvic floor dysfunction.

Previous pessary trials, where women are randomised prior to pessary fitting, have an attrition rate of approximately 40% [26, 33, 34]. A particular strength of the TOPSY trial design is to ensure that women have managed to retain their pessary for at least 2 weeks before they are eligible to be randomised. It is anticipated that having less attrition will support a true test of whether or not self-management is more effective in improving women’s quality of life than clinic-based care.

If the TOPSY study concludes self-management has a positive effect on a women’s quality of life in regard to management of pelvic organ prolapse and has cost benefits to the NHS, it is hoped that the intervention package (including the training manuals and literature developed for TOPSY) will be rolled out and implemented across the UK.

## Confidentiality {27}

All investigators and study centre staff involved in this study will comply with the requirements of the General Data Protection Regulations (GDPR) and the Data Protection Act 2018 in regard to the collection, storage, processing and disclosure of personal information.

Data collected during the course of the research is kept strictly confidential and accessed only by members of the research team and may be looked at by individuals from the sponsor organisation or NHS sites where it is relevant to the participant taking part in this trial.

## Trial status and internal pilot

The first participant was randomised on the 16th of May 2018. All six pilot centres were recruiting by June 2018, and 72 participants were recruited by the end of the pilot phase on 16 November 2018. The principal stop/go criterion was therefore met and the trial continued.

The secondary target of at least 60% of women self-managing at 2 weeks was also met with 83% of those randomised to self-management still self-managing at 2 weeks. The target of 20% of existing users was exceeded with 22% of eligible existing users randomised. The target of 40% of new users was not met with only 28% of eligible new users randomised. This lower than the anticipated figure led to a re-profiling of recruitment. As a consequence, the number of centres was increased, currently 21 centres are open to recruitment across the UK, and the recruitment period was extended until January 2020. Data collection will continue until 2021. As part of the process evaluation, data was gathered about participant recruitment processes, the findings of these elements of the pilot study will be submitted for publication imminently.

## Supplementary information

**Additional file 1.** SPIRIT 2013 Checklist: Recommended items to address in a clinical trial protocol and related documents.
